# Vitamin D and Allergy Susceptibility during Gestation and Early Life

**DOI:** 10.3390/nu13031015

**Published:** 2021-03-21

**Authors:** Daniela Briceno Noriega, Huub F. J. Savelkoul

**Affiliations:** Cell Biology and Immunology Group, Wageningen University & Research, 6708 WD Wageningen, The Netherlands; danibri@gmail.com

**Keywords:** vitamin D, allergy, neonate, early life, immune system, Treg, dendritic cells, anti-microbial peptides, gut barrier, respiratory infections

## Abstract

Worldwide, the prevalence of allergies in young children, but also vitamin D deficiency during pregnancy and in newborns is rising. Vitamin D modulates the development and activity of the immune system and a low vitamin D status during pregnancy and in early life might be associated with an increased risk to develop an allergy during early childhood. This review studies the effects of vitamin D during gestation and early life, on allergy susceptibility in infants. The bioactive form of vitamin D, 1,25(OH)2D, inhibits maturation and results in immature dendritic cells that cause a decreased differentiation of naive T cells into effector T cells. Nevertheless, the development of regulatory T cells and the production of interleukin-10 was increased. Consequently, a more tolerogenic immune response developed against antigens. Secondly, binding of 1,25(OH)2D to epithelial cells induces the expression of tight junction proteins resulting in enhanced epithelial barrier function. Thirdly, 1,25(OH)2D increased the expression of anti-microbial peptides by epithelial cells that also promoted the defense mechanism against pathogens, by preventing an invasive penetration of pathogens. Immune intervention by vitamin D supplementation can mitigate the disease burden from asthma and allergy. In conclusion, our review indicates that a sufficient vitamin D status during gestation and early life can lower the susceptibility to develop an allergy in infants although there remains a need for more causal evidence.

## 1. Introduction

In the last decades there is a widely recognized increase in the prevalence of allergies, especially in Western countries, including the United States, Europe and Australia. According to the Europrevall project, approximately 17 million people in Europe suffer from allergies, representing around 2.5% of the Dutch population, this percentage of prevalence holds true for most other European nations [1]. Among young children, the prevalence of clinical manifestations of allergy has doubled over the last decades [2]. Along with the increased prevalence of allergies comes a predictable increase in health care costs; in Europe alone the cost of allergy diagnosis and treatment have been estimated to be more than 25 billion euros per year [3]. Since allergic diseases can cause an array of symptoms ranging from itchy eyes or skin to diarrhea and in some severe cases anaphylaxis, the impact of individuals lives and of the health care system is of great concern. It was estimated in the State of the World Allergy Report from 2008, that in developed countries about 25% of the population has developed some clinical manifestation of allergy. This rise will lead to increased losses in productivity with absences increasing due to allergy symptoms both at school and at work, resulting in greater costs to society added to the already increasing health care costs [4]. Under these circumstances it is imperative that the scientific community works towards finding the cause or causes explaining this worldwide increase of allergies. 

Since the 20th century, also the prevalence of vitamin D deficiency has increased [5], whereby deficiency is commonly defined as serum 25-hydroxyvitamin D [25(OH)D] levels <20 ng/mL, with 20–30 ng/mL (or 800-1200 international units, IU) considered to reflect insufficiency. These values are based on modulation of calcium metabolism but still need to be defined for the capacity of vitamin D to modulate the development and activity of the immune system. The current guidelines generally recommend a steady state plasma level between 40–60 ng/mL [6,7]. When analyzing the vitamin D status, a serum concentration of less than <30 ng/mL was found in 54% of the pregnant women and in 75% of neonates. The authors suggested that maternal and new-born 25(OH)D concentrations are highly correlated and concluded that it should be a global priority addressing vitamin D deficiency in this population, since this deficiency during gestation and early life can lead to the development of an immature immune system in neonates, which increases the risk of developing allergies [8]. Cord blood vitamin D status generally was about 60% of the maternal level and at birth half of the infants had vitamin D level <50 nmol/L (or 800 IU/L). During follow-up, most of the children will acquire sufficient plasma levels of vitamin D, most likely due to the use of supplements. Bioactive vitamin D [1,25(OH)2D] exerts effects on cells of the innate and the adaptive immune system, and is therefore referred to as an immunomodulator [9]. Several immune cell types, including dendritic cells (DCs), macrophages, and T- and B-cells, are affected as they express a vitamin D receptor (VDR). Tissue resident dendritic cells (DC) respond to vitamin D and thereby affect the differentiation of T-cells into the different subsets to generate the necessary protection against pathogens [10,11]. Through the binding of bio-active 1,25(OH)2D to the VDR, vitamin D can influence the allergen induced inflammatory reaction [5]. Recently, two studies described an important role of vitamin D in clinical manifestations of asthma and immune function [12,13].

**Figure 1:**
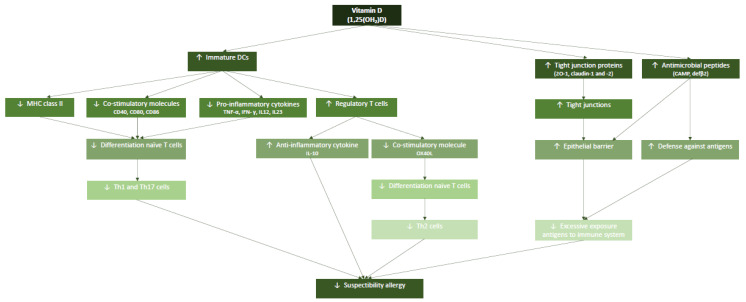
Schematic view of the specific effects of vitamin D on the neonatal immune system which are relevant to decrease the risk of developing an allergy against foreign food allergens.

The aim of this review is to study the effect of deficiency in vitamin D during gestation on the development of the neonatal immune system and its impact on early life, which can result in the development of an allergy in childhood (for an overview see Figure 1). We will first introduce relevant aspects of the immune system and its responsiveness on which vitamin D impacts and describing the underlying mechanisms of action. Next, we will describe aspects of vitamin D as an immunomodulator which impacts on the development of allergy in early life.

## 2. Fetal and Neonatal Immune Systems

In utero the fetus is exposed to a near-sterile environment, while during the birth process becomes confronted to a high microbial burden [14]. This sudden exposure to microbes affects the development and function of immune cells. Due to changes on the gene level but also epigenetically imprinted alterations the immune reactivity at neonatal age is different from that at later age. The innate immune system is comprised of physical barriers (epithelia in the gastro-intestinal and respiratory tract) and chemical barriers (anti-microbial substances) as well as cell different cell types like macrophages, DC, Natural Killer (NK) cells, and mast cells. The subsequent innate immune response after phagocytosis of infectious material ensures a balance due to the secreted pro-inflammatory (tumor necrosis factor (TNF)-α, interleukin (IL)-1β, IL-6, IL-12) and anti-inflammatory (IL-10, TGF-β) cytokines depending on the tissue micro-environment [15]. Upon birth, the innate immune system induces a state of immunotolerance to allow the fetus to co-exist in utero [16]. The survival of the neonate depends on mechanisms to inhibit the induction of inflammation although the fetal immune system is still able to mount an effective immune response to infections and antigen exposure after birth. 

The immune system of the mother detects the fetus as non-self due to the fact that the fetus has cells that bear both major histocompatibility complex (MHC)-molecules from the mother and the father. Consequently, the immune system of the mother reacts by producing Th1 lymphocytes which are involved in tissue rejection. As a result, there is a Th lymphocyte balance in the body of the mother that is skewed towards Th1 cells. Accordingly, the fetus protects itself against tissue rejection by producing Th2 lymphocytes at the connecting point between the fetal and maternal part of the placenta. The fetus will also be exposed to prostaglandin E2 (PGE2), IL-4, and IL-10 produced by the placenta which further stimulate the production of these local fetal Th2 lymphocytes. As a result, the balance of Th1/Th2 lymphocytes in the blood serum of the fetus is now skewed towards Th2. Finally, Th2 skewing in the infant continues up to a few months after birth. These Th2 type responses in the fetal and neonatal phase suppress inflammation and promote tolerance. Neonatal antigen-presenting cells (APC) including macrophages and DC are characterized by a lower expression of MHC class-II molecules along with a decreased expression of co-stimulatory molecules like CD80/CD86 as well as of Toll-like receptors (TLRs), making neonates more vulnerable to infections [15]. By 9 weeks of gestation, B lymphocytes develop in the liver and by 12 weeks they can be found in the bloodstream and spleen; while progenitor T-cells that migrate to the thymus at 7 weeks of gestation and begin to differentiate into CD4 and CD8 cells that carry α,β T-cell receptors [17]. However, there is a lack of development of secondary lymphoid tissue which most likely is attributed to the lack of antigenic stimulus [18]. During fetal and early life development Th1 cell-mediated immunity is immature due to a shift toward Th2 cell response since the IL-12 and type I interferon (IFN)-α/β producing DCs display a delayed maturation [19]. During gestation, fetuses acquire functional DCs by 13 weeks [20]. In the fetal period DCs produce IL-18 but this cytokine appears non-functional and therefore NK cells can’t be activated and remain in an immature phenotype, resulting in an impaired ability to kill infected cells [21]. In addition, the production of cytokines like IL-4, IL-5 and IFN-γ is suppressed in neonatal CD4^+^ T cells at neonatal age and therefore these T-cells react poorly to stimulation [22]. This impaired functioning might be due to limited function of intracellular signaling transduction pathways, low numbers of effector and memory cells (resulting in low levels of cytokines like IL-4 and IFN-γ), and the formation of immature DCs characterized by a reduced production of IL-12 [23]. At neonatal age also CD8^+^ T-cells show a decreased production of perforin, which is a glycoprotein responsible for pore formation, thereby limiting their cytolytic ability for virus-infected cells and tumor cells [21]. 

Studies in umbilical cord blood suggest that functionally mature CD4^+^CD25^+^ Tregs are present but carry a naïve phenotype, and playing a potential immunoregulatory role in the fetal period [24]. It is important to understand that in the fetal period regulatory cells are a secondary line of defense by suppressing potentially harmful autoimmune responses. The characteristic transcription factor FoxP3 (Forkhead box P3) induces the differentiation of naive T cells into CD4+CD25+Foxp3+ Tregs and the expression is upregulated by exposure to TGF-β and thereby maintaining peripheral tolerance [25,26,27].

When analyzing the humoral immune response, it was observed that T cell-independent antibody responses which are commonly specific for polysaccharide antigens, start to become functional after the third month of pregnancy. Only at 4 to 5 years of age these responses reach adult levels when the marginal zones in the spleen become fully mature and at that time also the expression of CD21 reaches adult levels on the B-cells [28]. Also the T cell-dependent response capacity, which is generally directed at protein antigens, is not yet fully mature at early age which is due to several reasons, including the low expression of CD40 ligand on activated cognate CD4+ Th cells resulting in an inefficient priming of the primary antibody response, the immature status of DC in the follicles in spleen and lymph nodes, the low degree of somatic hypermutation in the B-cells resulting in very limited affinity maturation, and the limited support of growth factors from the stromal micro-environment [29]. Nevertheless, stimulation of B-cells through TLR-9 can partially compensate as this signaling induces B-cell differentiation into plasma cells secreting immunoglobulin (Ig)M antibodies [30].

As a result the neonatal immune response capacity of T and B-cells is very limited with decreased numbers of Treg and failure to induce effective oral tolerance [31]. This puts the fetus and the neonate at high risk to develop infectious diseases. In addition, nutritional deficiencies have a large impact as the immune system is energy-demanding. The interaction between the developing immune competence and the nutritional status impact on early life health and development of disease, including allergy [32,33].

## 3. Neonatal Gut-Associated Mucosal Immune System

At birth, colonization of the gut with commensal bacteria provide antigens that contributes to the proper development of the gut-associated lymphoid tissue (GALT). This includes the expression of genes important in the development of effective immune responses in the terminal ileum [34]. In germ-free mice, the absence of gut microbiota results in a disturbed intestinal and immune development, accompanied with the generation of immune deficiencies [35]. These immunodeficiencies might be based on decreased presence of important anti-microbial molecules like angiogenin-4, RegIIIγ, sIgA, adenosine triphosphate (ATP), MHC class III, toll-like receptor (TLR) 9, and IL-25 [36]. These germ-free mice will contain fewer numbers of immune cells, including IgM and IgA secreting plasma cells, in the various compartments of the GALT, including the Peyer’s patches (PPs), lamina propria (LP), germinal centers (GCs) and isolated lymphoid follicles. Secretory IgA antibodies are the most abundantly produced antibody class and in the gut they provide protection against aberrant microbial stimulation by pathogens. In addition, activated CD4 and CD8+ T-cells are reduced in the lamina propria but also in the mesenteric lymph node. In particular this holds true for Foxp3+ Treg which results in enhanced susceptibility to develop chronic low-grade inflammation [37].

The process of induction of tolerance is crucial in a developing fetus to prevent the development of a damaging maternal inflammatory status that might result in miscarriage or fetal resorption. This is achieved by the induction of increased numbers of functional Treg, suppressing maternal T-cell proliferation and Th1-mediated rejection responses [26]. Towards delivery, the numbers and activity of Treg start to decline, reaching levels similar to adults at birth [24]. This is particularly relevant in the gut as this is the place where, after birth, the majority of antigens will be present and due to their innocent behavior need to be tolerated rather than inducing immune responses. In addition, the B1 or CD5+ B-cell subset are able to secrete IL-10 contributing to the immunosuppressive fetal environment. These B1 cells also produce low-affinity and highly cross-reactive IgM and IgA antibodies, they provide protection against infections and are abundantly present in the neonatal gut mucosa with high numbers at birth (up to 40% of B-cells) and increasing further up to 4 months of age [27]. They subsequently decline with the concomitant decrease in gut permeability [26,29]. The development and function of the immune system at fetal and neonatal age is further regulated by myeloid-derived suppressor cells (MDSC) that are strongly associated to the inflammatory status in the gut [38,39,40]. MDSCs mainly suppress Th1 cell activation and proliferation by inhibiting the release of IL-5, IL-17, and IFN-γ [37]. As a result there is a preferential development of Th2-mediated responses and inhibited cytotoxicity of NK cells [38]. The immune system is further regulated by mesenchymal stromal cells (MSC) in the intestinal lamina propria [41,42,43]. MSC suppress the differentiation of immature DC [21], the generation and function of naïve and memory CD8+ T-cells and NK cells [38]. Also these MSC promote the generation of functional Foxp3^+^ Treg cells. Collectively, this suppression of the immune system of the fetus, prevents chronic inflammation and promoted successful gestation and continues for about 4 months after delivery.

## 4. Vitamin D

Vitamin D is a fat-soluble vitamin displaying immunomodulatory effects on immune cells and is implicated in the development of allergy [9,12]. DC and T cells are modulated by vitamin D to achieve tolerance while also suppressing inflammation by limiting the innate immune response to antigens including allergens [44,45,46]. While many of the reported immune-related activities of vitamin D are well-described, they remain mainly observational and it remains to be established whether there is causal role in the induction of anti-inflammatory activity in immune-mediated diseases, including allergy [47,48,49,50]. In early childhood, a deficient vitamin D status correlated with the presence of food allergy, while higher plasma levels of vitamin D correlated to lower levels of C-reactive protein (CRP), IL-6 and TNF-α, indicating a lower degree of inflammation. Despite the fact that these data were obtained from observational studies, it is increasingly suggested that vitamin D sufficiency limits the development of inflammation [50].

### 4.1. Vitamin D Synthesis

Vitamin D is obtained via exposure of the skin to sunlight or via the diet in foods such as oily fish, eggs and liver. While about 80–90% of the required daily dose of vitamin D is received via sun exposure, while the remaining 10% is provided via the diet [51,52]. Vitamin D can be divided into two types: D2 and D3. Vitamin D2 or ergocalciferol is produced in yeast and plants when UV light acts on ergosterol. In humans, vitamin D3 is known as cholecalciferol, produced by the conversion of endogenous 7-dehydrocholesterol through a photochemical reaction when the skin is exposed to solar UV-B irradiation (mainly by the wavelengths 290–320 nm). Dietary or exogenous sources of D3 are limited to oily fish such as: salmon, mackerel, sardines, tuna plus fish liver oil, egg yolk, as well as vitamin D-fortified foods and supplements. In adults, vitamin D3 is considered preferable to vitamin D2 in its capacity to sustain adequate plasma levels of 25-hydroxyvitamin D (25OHD), as D2 has a lower binding affinity for the chaperone protein vitamin D binding protein (VDBP) and will therefore be cleared more rapidly from the circulation than 25-hydroxyvitamin D3 [53]. Both vitamin D2 and D3 give rise to the formation of the bioactive form of vitamin D, 1,25-dihydroxyvitamin D (1,25(OH)2D). To describe amounts of vitamin D, different units of measurement are used internationally, including for doses 1 microgram (μg) = 2.5 nanomoles (nmol) = 40 international units (IU) of cholecalciferol, while concentrations of 25OHD are reported in 1 ng/mL = 1 μg/L = 2.5 nmol/L [54].

There are feedback loops that control the production and amount of 1,25(OH)2D. CYP27B1 converts 25OHD to 1,25(OH)2D and this activity is essential to stimulate the gene expression of antimicrobial peptides (AMP) such as cathelicidin and β2-defensin by Paneth cells in the bottom of the crypts in the gut epithelium [55]. AMP contribute to protection of the epithelial barrier by killing many pathogens and modulating the secretion of cytokines [56]. The AMP cathelicidin is chemo attractive for neutrophils and monocytes that phagocytose microbes, and it stimulates subsequent microbial killing. These activities are dependent on the presence and activity of 1,25(OH)2D. A feedback mechanism was proposed that prevents toxicity by high plasma levels of vitamin D in which 1,25(OH)2D induces the expression of the CYP24 enzyme that degrades both 25OHD and 1,25(OH)2D to generate biologically inactive metabolites [57]. The plasma levels of 1,25(OH)2D are controlled by the vitamin D-activating hydroxylase CYP27B1 activity in the kidney [58], but also immune cells, including macrophages and DCs, express CYP27B1. By converting 25OHD into 1,25(OH)2D, while lacking a feedback mechanisms this allows for production of high local concentrations of 1,25(OH)2D3 [59]. This condition refers to plasma levels in the range of 10–50 ng/mL (25–125 nmol/L) requiring an intake of about 40–60 μg per day [54,60]. 

Vitamin D intake can thus determine the vitamin D status in the plasma. However, vitamin D supplements usually contain higher doses of vitamin D compared to the levels of the vitamin present in foods. As a result, such supplements effectively elevate plasma levels of 25OHD at a rate of 5 nmol/L per supplied 100 IU/day [61]. Supplementation with 1000 IU a day will bring 50% of the population to adequate levels, while an oral intake of 2200 IU of vitamin D may be necessary to achieve an adequate status of vitamin D. In a RCT with pregnant women, daily cholecalciferol supplementation starting before 20 weeks of gestation with 400 IU/day vs. 2000 IU/day, was analyzed for its effect on markers of immunoregulation [62]. At 36 weeks of gestation, the group receiving 2000 IU/day of vitamin D showed a larger (*p* < 0.0001) increase in the plasma levels of 25OHD (81.1 nmol/L to 116 nmol/L) compared to the group receiving 400 IU/day (69.6 nmol/L to 85.6 nmol/L). The high dose group showed more IL-10 producing Treg cells (*p* < 0.007), indicative of a better regulation of inflammation during pregnancy [62]. These studies underscore that vitamin D supplementation in adequate doses show protective properties in pregnancy, and these are probably mediated by the immunoregulatory role of vitamin D.

### 4.2. The 1,25(OH)2D-VDR Complex and Genetic Polymorphisms

The Vitamin D receptor (VDR) is abundantly expressed in different tissues and cells, including most immune cells, including monocytes, macrophages, DCs, T cells and B cells. Bioactive 1,25(OH)2D, carrying a steroid backbone is able to diffuse through the cell membrane to interact with the cytoplasmic VDR and this receptor-ligand complex heterodimerizes with the retinoid X receptor (RXR) and the combined VDR-1,25(OH)2D-RXR complex then acts as a transcription factor moving into the nucleus and regulating the expression of a collection of target genes [57]. The promotor region of these target genes contains vitamin D responsive elements (VDREs). As a final result, VDR-1,25(OH)2D modulates the expression of target genes and regulates inflammatory immune responses [63].

The susceptibility to development an allergy has a genetic background and the heritability is dependent on many different genes and proteins, although their individual contributions are still poorly understood. Specific mutations and/or single nucleotide polymorphisms (SNPs) in selected genes were shown to influence their expression and thereby their role in the development of clinical manifestations of allergy [64,65]. Analysis of association between certain SNPs and their allelic forms enable the identification of those genetic factors that are involved in the predisposition to allergy. Such studies identified genes involved in vitamin D metabolism as promising candidates of risk factors to be used in genomic marker analysis. These genetic studies linked vitamin D metabolism to the development of allergy in early life. 

Both expression and nuclear activation of VDR are necessary for proper downstream responses induced by vitamin D. By using different types of restriction enzymes in RFLP (Restriction Fragments Length Polymorphism) analysis of DNA from vitamin D treated allergic individuals different genetic variations in VDR, known as polymorphisms, were identified [66]. In childhood asthma, vitamin D levels act as a possible marker for disease severity via the VDR Apal A/a genotype which was positively associated to well-controlled asthma [67]. Additional polymorphisms such as Taql, Bsml and Fokl have also been identified as contributors to asthma susceptibility [68]. These findings offer supporting evidence to the relevance of vitamin D in asthma. 

The plasma vitamin D half-life in free form is very short, but the half-life is extended by the binding to the chaperone vitamin D binding protein (VDBP)which enables the transport to target tissues [69]. Thus, mutations in the VDBP gene may be the cause of decreased level of vitamin D in plasma [63]. In addition, these mutations correlated with differences in 25(OH)D3 or VDBP concentrations in plasma [70]. One of the most common genetic variants in VDBP gene are on chromosome 4q13 is polymorphism in exon 11 (SNP rs7041), resulting in a thymine (GAT) transversion into guanine (GAG), which changes an aspartic acid (Asp) into glutamic acid (Glu) in polypeptide chain [71]. The SNPs rs7041 is the most important SNP that together with SNP rs4588 explains almost 80% of the variation in VDBP [72,73]. Despite differences in the degree of polymorphism in the VDBP gene often individuals carrying these SNPs, showed no difference in plasma levels of vitamin D. Apparently, the interaction between the bio-active 1,25(OH)2D and the receptor, more than VDBP, determines the final effect as we also hypothesized in our analysis of the vitamin D metabolism in autistic children [74]. Alternatively, Although SNPs in both CYP27B1 and CYP24A1 genes were identified they are quite rare in different populations and therefore their effect remain largely unknown [75].

## 5. Immunomodulation by Vitamin D

### 5.1. Vitamin D3 Inhibits the Maturation of DCs

**Figure 2:**
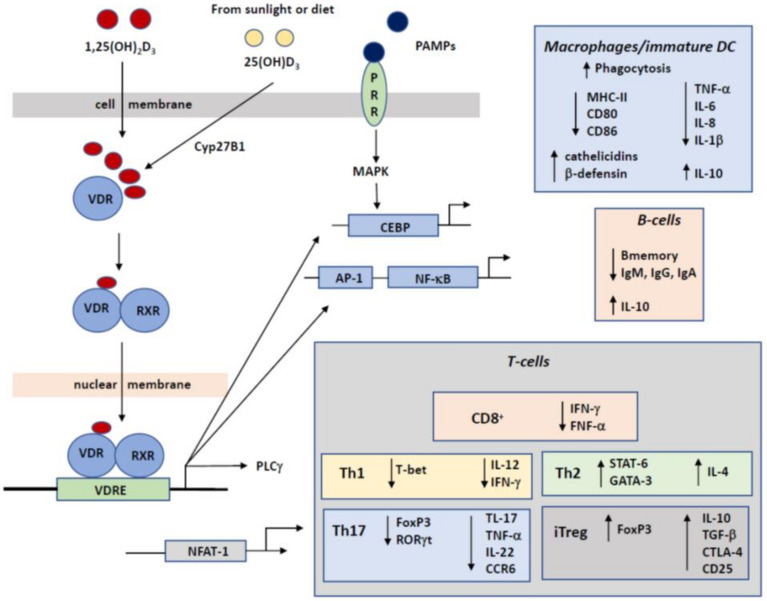
Schematic view of the signaling transduction cascades upon vitamin D interaction with immune cell and their consequences for gene transcription profiles for the different important immune cell populations.

The prime function of vitamin D is regulating calcium homeostasis and it is therefore important for bone health. Here we focus on the capacity of vitamin D to affect the development and functioning of the immune system (Figure 2). The binding of 1,25(OH)2D to the VDR may reduce the differentiation of naive T cells into specific Th subsets, like Th1 and Th17, while promoting the differentiation of Th2 and Treg. Vitamin D is thus known to modulate the balance between these different T-cell subsets which may contribute to the development of allergy. Since at young age, most T-cell populations are still naïve rather than belonging to the memory pool, this effect of vitamin D will be more apparent in driving the development of allergy. This immunomodulatory effect of vitamin D on T0-cell subset differentiation is mediated through its effect on dendritic cells, as these cells are the prime type of antigen-presenting cells (APCs) that drive T cell differentiation into effector cells with pro- or anti-inflammatory properties. Since 1,25(OH)2D inhibits maturation of DC and keeps them in a more immature but tolerance-inducing phenotype, this modulation of DCs is crucial in initiating adaptive immune responses and maintenance of self-tolerance. These immature DC are functionally and phenotypically different but active in immunoregulation [76].

A results of the 1,25(OH)2D induced immunomodulation of DC is the altered cytokine production profile [77,78,79,80,81]. In a DC cell culture treated with 1,25(OH)2D, the production of IL-12 and IL-23 was decreased while the production of IL-10 and TNF-α was increased, resulting in a diminished differentiation of naive T cells into Th1 and Th17. The increased production of IL-10 was based on the increased generation of Tregs by immature DCs [80]. Tregs produced IL-10 which promotes induction of tolerance and contributes to the adaptive immune homeostasis. This resulted in a shift from a Th1 and Th17 mediated inflammatory immune response towards a Treg response [77,78]. The secreted IL-10 is considered an anti-inflammatory cytokine, as it reduces the development of inflammation and inflammatory immune responses. Moreover, IL-10 exerts a pro-apoptotic effect on mature DCs [82]. Due to the reduction in mature DCs fewer naive T cells will differentiate and become activated. In this way, the reduced number of mature DCs induced tolerance.

### 5.2. The Binding of 1,25(OH)2D Lowers the Expression of MHC II and Co-Stimulatory Molecules

Many tissue and cell types contain enzymes that metabolize vitamin D from cholecalciferol to the active calcitriol. Besides, about 2000–8000 VDR binding sites are present per target cell thereby adding to the heterogeneity of the vitamin D response in different target cells [83]. Immature DCs can be identified by the low expression of MHC class II molecules and also of the CD40 and CD80/CD86 co-stimulatory molecules [80,82]. The result of a low expression of MHC class II is the decreased capacity to to present antigenic peptides to antigen-specific but naive T cells. As a consequence the reduced differentiation of naive T cells into Th1 or Th17 cells was also reduced. This effect is amplified by the decreased expression of the co-stimulatory molecules CD40, CD80 and CD86. 

Interestingly, Barragan et al. identified that the expression of the co-stimulatory molecule OX40L was reduced on DCs treated with 1,25(OH)2D [45]. A high expression of OX40L is present on mature DCs enabling the differentiation of naive T cells into Th2 cells. Moreover, the results showed that the increased amount of Tregs also suppressed the expression of OX40L on DCs. Because of the reduced expression of OX40L, also the generation of Th2 cells was suppressed thereby reducing the development of a Th2 reaction against antigens, including allergens. 

### 5.3. The Role of Glucose on the Binding Response of VDR-1,25(OH)2D on DC and T-Cells

In DC the binding of 1,25(OH)2D to the VDR inhibited the maturation resulting in immature DCs. Interestingly, glucose was found to promote this inhibiting effect thereby contributing to the tolerogenic phenotype [78,84]. In monocyte-derived DCs, treated with 1,25(OH)2D in the presence of glucose, the typical tolerogenic cell surface marker expression was observed characterized by a decreased expression of co-stimulatory molecules and MHC class II, and an increased production of IL-10. However, when these DCs were treated with 1,25(OH)2D in the presence of limited glucose, a lower number of immature DCs was observed together with increased expression of co-stimulatory as well as MHC class II molecules, but lower levels of IL-10. Therefore, both studies showed that the availability of glucose could be important for the effect of VDR-1,25(OH)2D to induce and maintain immature DCs. Additionally, Vanherwegen et al showed that 6-phosphofructo-2-kinase-4 (PFKFB4), a bifunctional glycolytic enzyme that activates phosphofructokinase-1 (PFK-1) was an important metabolic sensor in glycolysis, and acted as a direct transcriptional target for 1,25(OH)2D which contributed to the induction of immature DCs [78]. Since PFKFB4 regulates glucose metabolism via PFK-1, its function in the binding of 1,25(OH)2D to DCs could be important for the induction of immature DCs. 

Despite that the exact mechanism and consequences of the binding of 1,25(OH)2D to VDR in immune cells like DCs is still unclear, the results of these studies suggested that the availability of glucose and the presence of PFKFB4 could be important for the cellular response and contributed to the induction of immature DCs. These immature DCs are more tolerogenic as described above. With this tolerogenic phenotype of immature DCs, 1,25(OH)2D downregulated the antigen-presentation capacity, co-stimulation and secretion of pro-inflammatory cytokines of DCs. As a result, 1,25(OH)2D induced a decreased differentiation of naive T cells by DCs. On the other hand, the induced immature DCs upregulated the amount of Tregs and IL-10, which induced tolerance. Studies using VDR knockout mice showed that 1,25(OH)2D binding to VDR mediates homing of activated Treg-cells to the gut, which results in low systemic levels of IL-10 as well as an increased global inflammatory responses in these mice [85]. These Tregs also suppressed the expression of OX40L which in turn suppressed the development of Th2 cells. Therefore, immature DCs could induce a tolerogenic Treg response and prevention of a Th1 and Th17 inflammatory mediated immune response or a Th2 response against allergens based on the regulation of their glucose metabolism. Due to these processes, 1,25(OH)2D may reduce the development of an allergy.

### 5.4. Vitamin D Induces the Expression of Anti-Microbial Peptides in the Gut

Anti-microbial peptides (AMP) contribute to the protective mechanism of the epithelial barrier. AMPs can kill pathogens which in turn lowers the penetration of pathogens. Consequently, the development of an excessive immune response against large amounts of antigens is reduced. Therefore, it was suggested that AMPs are important in killing a broad range of pathogens [86]. Besides, 1,25(OH)2D induces the expression of AMPs and thereby contributes to the development of allergies [87,88,89]. The two important microbial peptides are cathelicidins and β2-defensin, and these are the final result of epithelial cells exposed to 1,25(OH)2D that complexes with VDR and heterodimerizes with RXR that subsequently bind to the VDRE in the DNA. Via the VDRE present in the promotor of anti-microbial genes, 1,25(OH)2D can induce the expression of these AMPs. The increased expression of these AMPs protects against the penetration of pathogens by killing them, this prevents an excessive penetration of pathogens. In this manner, AMPs prevents the induction of an excessive immune response against antigens [90]. Therefore, the studies showed that an increased amount of AMPs contributed to the reducing risk to develop an allergy.

### 5.5. 1,25(OH)2D Decreases the Mucosal Permeability

It was described that 1,25(OH)2D is important for an efficient barrier function in the gut [78,91,92,93,94,95]. Tight junctions (TJs) consist of different proteins that interact with partner proteins on the neighboring cell to ensure that the intestinal epithelial cells are strongly and closely hold together to form a well-functioning barrier. 1,25(OH)2D has the capacity to increase the expression of TJ proteins, like ZO-1, claudin-1 and claudin-2 [91,92,93,94,95] and thus enhances the epithelial layer by decreased the permeability. Upon vitamin D deficiency, disruptions in the mucosal barrier and increased permeability, also called a leaky gut, were found in affected individuals [96]. Inflammation in the gut associated with a leaky gut and other inflammatory gut disorders, will compromise the absorption of fat leading to a decrease in systemic levels of vitamin D. Moreover, multiple studies reinforced that vitamin D deficiency also resulted in a reduction in the expression of the TJ proteins ZO-1, claudin-1 and claudin-2 [92,93,94,95]. As a result, the immune system was exposed to an increased number of antigens, including allergens, which stimulated an excessive immune response increasing the risk to develop an allergic reaction against antigens [97,98]. If the expression of the TJ proteins was reduced due to a deficient vitamin D status, the mucosal permeability increased which resulted in an increased absorption of allergens and their exposure to the mucosal immune system and increasing the risk to develop an allergic reaction [99]. 

## 6. Vitamin D during Gestation and Early Life

### 6.1. Vitamin D in Early Life

Allergy and asthma are important chronic diseases at childhood age. Dietary exposure in utero and during infancy are considered important for the susceptibility to develop allergic diseases. In many Western populations where childhood allergies and asthma are common, it is common to have a high dietary intake of vitamin A in combination with a low intake of vitamin D. The intake of high doses of vitamin A can cause adverse health effects as many food products are reinforced with vitamin A. The sufficient intake of vitamin D can counteract this effect as both vitamins compete for the RXR binding [100]. A normal maternal vitamin D status during gestation is important for the new-born to develop an adequate and healthy vitamin D status. This can be achieved as 25(OH)D crosses the placental barrier and determines vitamin D levels of the new-born at birth. The fetus is exposed to low levels of vitamin D when the mother is vitamin D deficient during gestation, and thereby the fetus becomes functionally vitamin D deficient and therefore at birth will have a low vitamin D status [101]. 

In early life infants receive breastmilk from their mother and thus their resulting vitamin D status depends on the vitamin D content in breastmilk. When the maternal vitamin D status is still low after giving birth, the breastmilk will contain low levels of vitamin D and thereby contribute to a low vitamin D status of the infant [102]. Since it is recommended to give exclusive breastfeeding in the first six months of life, breastmilk is the only source for the infant to consume vitamin D. Besides this, due to the very sensitive skin of infants, limited sun exposure is recommended for infants. Consequently, infants cannot generate enough vitamin D from the sunlight [103]. In conclusion, the vitamin D status of infants is heavily dependent on the maternal vitamin D levels. After six months of age, breastmilk alone is not enough to meet the infant’s energy and nutrient requirements, which is why around this time solid food are introduced in the diet. To achieve proper vitamin D levels, recommendations comprise 600 IU vitamin D per day for the breastfeeding period and 400 IU vitamin D per day for infants [104,105]. During the prenatal phase and early life period, vitamin D can modulate the developing immune system and a disturbed vitamin D status in early life might be associated with an increased risk to develop an allergic disease [106]. 

The most common food allergy at childhood age is cow’s milk allergy with a prevalence of 2–6% of children [107]. Consumption of milk and dairy products during pregnancy significantly reduced subsequent cow’s milk allergy in the offspring (OR = 0.56), while also high levels of IgA antibodies present in cord blood provide protection against the development of food allergy. As described above, exclusive breast or infant formula feeding during the first 4 months of life is the only source of vitamin D. Unfortunately, vitamin D is only transferred to breast milk in limited amounts. To ensure sufficient intake of vitamin D, infant formula is fortified with 360–520 IU/L of vitamin D depending on the age of the infant. In addition, vitamin D supplementation is recommended for children up to 4 years of age, due to the limited exposure to sunlight and insufficient vitamin D levels in foods. The recommended dose of 10 μg vitamin D for children up to 4 years of age is below the safety level of 25 μg/day (1000 IU) even when taking a daily supplement containing 10 μg (400 IU) [108,109].

### 6.2. Vitamin D and Allergy

The relevance of vitamin D in the immune system is undeniable, and together with the association between the currently worldwide problem of deficiency and the rising numbers of allergic and asthmatic children is becoming widely accepted. Consistently, vitamin D deficiency has been associated with the risk of developing allergies [110,111,112]. In addition, 1,25(OH)2D inhibits maturation of DCs, increases IL-10 secretion in APCs and Foxp3+ Treg cells and reduces T cell activity [113]. It has also been shown that when anti-inflammatory activities of vitamin D are lacking, intensive responses of the immune system occur to allergen exposure by secreting large amounts of IgE antibodies and Th2 derived IL-4. Importantly, restoring the vitamin D status by supplementation will inhibit the plasma levels of TNF-α and as a result increase the expression levels of the VDR and the factor prohibitin, which together will reduce the allergic inflammation in skin and airways [114]. Up to 2007, it was hardly known that also in plasma cells 1,25(OH)2D binds to VDR, and this results in inhibition of production and secretion of antibodies, including allergen-specific IgE. However, these studies did not segregate whether the binding to VDR in plasma cells of 1,25(OH)2D inhibits the production of IgE, inhibits class-switching from IgG to IgE or that the differentiation of plasma cells is inhibited which result in a decreased production of IgE. There further studies elucidating the precise role of vitamin D3 in the production of IgE antibodies are urgently needed. The susceptibility to develop allergy originates at least in part in utero as allergic manifestations frequently occur within the first months after birth. In addition, immune responses at neonatal age differ between infants who later will or will not develop allergic disease. Vitamin D deficiency in perinatal life span is suggested to be causally related with modulating susceptibility to early allergic sensitization and subsequent development of clinical manifestations of allergic disease [115]. 

Apart from modulation of the functional responsiveness of immune cells, vitamin D also contributes to the stability and integrity of the epithelial barrier at the mucosal tissues in the gastrointestinal and respiratory tracts. All these features place vitamin D as an interesting option to be used as a complement to allergen-specific immunotherapy [116,117]. However, the relationship between plasma levels of vitamin D and clinical manifestations of allergy is not necessarily linear, as both low and high levels of vitamin D may correlate with increased levels of allergen-specific IgE antibodies. Many studies describe that in children vitamin D deficiency is associated with increased levels of allergen-specific IgE sensitization and risk for asthma development. On the other hand some studies indicate an increased risk for atopy and allergy development upon vitamin D supplementation in early [118].

Vitamin D and also metabolites derived thereof are widely known to have anti-inflammatory and antioxidative effects and regulate immunopathological inflammatory responses manifested at peripheral and central sites. The disturbed balance between pro and anti-inflammatory effects is based on the induction of anti-inflammatory activities rather than inhibition of pro-inflammatory effects. Based on clinical studies it is known that vitamin D modulates innate immune responses to pathogens [119], and vitamin D regulates, rather than inhibits, adaptive immune responses in different chronic inflammatory and autoimmune diseases, and vitamin D supplementation was shown to be effective in decreasing adverse effects of inflammatory diseases, including allergy [120,121,122,123]. Vitamin D is able to increase anti-microbial immune defense and it strengthens immune regulation and thereby ensures peripheral tolerance at mucosal tissues. In addition, vitamin D inhibits allergic inflammation by the generation of Treg. Collectively, these findings provide a strong case that already in early life vitamin D has the capacity to improve respiratory health and control of asthma development and also induces oral tolerance to food allergens. 

An important study showed that in cow’s milk allergic children with lower levels of vitamin D as compared to healthy children, the increased expression of Foxp3-mRNA predicted a faster development of oral tolerance. Moreover, vitamin D supplementation during pregnancy resulted in vitamin D sufficiency in the newborns and this reduced the incidence of asthma and/or recurrent wheezing by 3 years of age [124]. However, recently the same group reported now a lack of vitamin D efficacy when analyzing the same children at 6 years of age [125]. Nevertheless, it is plausible that vitamin D supplementation may decrease the susceptibility to viral infections during the first 3 years of life, and thus vitamin D supplementation during pregnancy may still play a role in averting wheezing in infants that for genetic reasons run a risk in the development of asthma-like symptoms especially in the virus season [126]. In these studies, mothers started vitamin D supplementation at weeks 10 to 18 of pregnancy, while as stated in the 2020 paper, lung development already starts at 3 to 4 weeks of gestation and therefore early vitamin D levels may still be important in this process. Moreover, as the authors point out a limitation of their study was that there was no postnatal supplementation provided and that the mothers included in the study were not stratified according to their initial vitamin D levels. Therefore, as the authors pointed out, prenatal vitamin D supplementation alone did not limit the development of asthma in these children at 6 years of age [125].

Using a mouse model of induced allergic asthma, 1,25 (OH)D3 treatment significantly reduced IL-5 and IL-13 production in bronchoalveolar lavage fluid, inhibited the formation of serum OVA-specific IgE levels, and augmented IL-10 secretion in lung tissue. Baris et al. studied the effect of subcutaneous immunotherapy (SCIT) using vitamin D as an adjuvant treatment on house dust mite sensitized asthmatic children [127]. The study compared the results of SCIT combined with vitamin D, SCIT on its own and pharmacological treatment alone. Both SCIT treatments were significantly more efficient than the administered anti-inflammatory drugs at reducing symptom and medication scores and increasing levels of antigen-specific IgG4 and IL-10 were observed in the plasma of these children. Although similar in their outcomes, there were some differences between the two SCIT treatments: the group that underwent SCIT together with vitamin D had lower asthma symptom scores, had a higher discontinuation rate of inhaled corticosteroid and higher FOXP3 expression levels than the cohort that received plane SCIT.

Vitamin D has also been combined with sublingual immunotherapy (SLIT). In a study aiming to evaluate the impact of vitamin D in allergic rhinitis, young children were given daily allergen tablets supplemented with vitamin D or with a placebo [128]. By regulating inflammation through inhibition of the activation of proinflammatory cells and their production of proinflammatory cytokines, vitamin D can modulate the risk and outcome of acute infections and the risk to develop inflammation-mediated diseases, including allergy [129]. The use of vitamin D proved safe and more effective in reducing nasal and asthma symptoms. In patients with allergic rhinitis the efficiency of SCIT was evaluated by measuring their circulating levels of vitamin D [130]. They observed a direct relation between reduction in symptom scores and the levels of serum vitamin D. The greatest symptom amelioration occurred in patients with “sufficient” vitamin D levels, while subjects suffering from vitamin D deficiency had a significantly smaller reduction in symptomatology. Despite SCIT showing to be able to improve symptom scores even in patients with vitamin D insufficiency, this study reveals how this hormone is important in mitigating the allergy reaction and suggests that vitamin D supplementation might be an interesting option to increase the effectiveness of ASIT.

## 7. Conclusions

The bioactive form of vitamin D, 1,25(OH)2D, has multiple effects on the immune system and can thereby contribute to mitigating the development of an allergy. Vitamin D can keep DCs in an immature state which is characterized by a decreased expression of MHC class II as well as co-stimulatory molecules. As a result, the differentiation of naive T cells into Th1 and Th17 decreases while these immature DCs upregulate Tregs and IL-10. This altered DC differentiation state induces peripheral tolerance. The increased amount of Tregs also suppresses the expression of co-stimulatory molecule OX40L which lowers the differentiation of naive T cells into Th2 cells. In this manner, the effect of 1,25(OH)2D on DCs contributes to a tolerogenic response against allergens and prevents the development of an allergic immune response. 

In addition, 1,25(OH)2D enhances the expression of the TJ proteins ZO-1, claudin-1 and clausin-2 resulting in the formation of TJs that increase the gut barrier function. This decreased epithelial permeability reduces an excessive exposure of allergens to the mucosal immune system thereby reducing the risk to develop allergy. Moreover, 1,25(OH)2D increases the expression of cathelicidins and defensins as AMP via the binding to VDRE which is present in the promotor of these anti-microbial genes. The increased amount of AMP enhances pathogen killing preventing the exposure to excessive amounts of antigens to the immune system and additionally reducing the risk of developing an allergy.

It can be concluded that obtaining and maintaining an adequate vitamin D status during gestation and early life is important to develop a tolerogenic immune reactivity against allergen exposure. Hence, a sufficient amount of the bioactive form of vitamin D has important supporting effects to develop a proper working and tolerogenic immune system. Therefore, adequate levels of vitamin D during gestation and early life could reduce the risk to develop an allergy. For an overview of the effects of 1,25(OH)2D on important immune cell populations refer to Table 1. This review provides an overview of the clinical and the experimental evidence that highlights that sufficient vitamin D levels during pregnancy can modulate inflammatory processes like allergy. This conclusion is supported by various experimental and observational studies. The underlying mechanisms explaining this activity are consistent, fit into the current insights on immunoregulation and control of tolerance and inflammation, and provide available options for the prevention and management of manifestations of allergy in early life. However, as observational studies only provide a limited level of evidence given their sensitivity to bias and their inherent inability to establish causation. So far, randomized controlled trials (RCTs) and systematic reviews of RCTs have focused on specific population groups, such as allergic children. There remains a need for systematic reviews on RCTs on different populations, including those at risk to develop allergic sensitization and subsequent development of clinical manifestations of allergy.

## Figures and Tables

**Table 1:** Immune system characteristics in fetal and neonatal age and effects of vitamin D supplementation.

	Immune System		Suppletion Vitamin D	Immune Health Effect	Ref.
	Fetal	Neonatal			
**Innate**					
Cathelicidin:	high	low	high	anti-microbial peptides	[88,131]
Β-defensin:	High	low	high	neutralization toxins	[88,131]
DC					
IL-12, IFN-α/β:	low	high	low	inflammation	[18,19,80,82]
**Macrophages**					
ROS, CD14+:	low	high	low	hyperinflammation	[40,132]
NK cells:	low	high	high	Killing infected cells	[21]
MDSC:	high	low	low	immunosuppression	[132]
Th1, Th17:	low	high	low	inflammation	
Th2:	low	low	low	Allergy, tolerance	[15]
CD8-cells:	low	high	high	Killing infected cells	[85]
Treg:	low	low	high	Tolerance	[31,62,85]
**B-cells**					
Antibodies	low	high	low	No autoantibodies	[133]
Breg	low	high	high	tolerance	[133]

## Data Availability

Not applicable.
